# Hysteria in neurology: a diagnostic approach to conversive disorder

**DOI:** 10.1192/j.eurpsy.2022.1000

**Published:** 2022-09-01

**Authors:** A. Sanz Giancola, I. Cuevas Iñiguez, C. Alvarez Garcia, M.D.C. Molina Liétor, M. Blanco Prieto

**Affiliations:** Hospital Universitario Príncipe de Asturias, Psychiatry, Alcalá de Henares, Spain

**Keywords:** neurology, conversive disorder, diagnostic, hysteria

## Abstract

**Introduction:**

Conversion disorder (a term that describes what was previously called hysteria) refers to motor or sensory symptoms, or both, that resemble a neurological disease, but that do not originate from or cannot be explained by a known physical disease.

**Objectives:**

To find reliable tools that can guide the difficult diagnosis of conversion disorder.

**Methods:**

Bibliographic review

**Results:**

The exact prevalence of the disorder is unknown. It is estimated that approximately 5% of referrals to neurology are for this disorder. Approximately one third of patients referred to the neurologist have symptoms that cannot be explained by an organic disease. Involuntary movements are the most common motor manifestations of the conversive syndrome, being tremor one of the most frequent manifestations. The first differential diagnosis of conversion disorder is neurological disease. It is currently not necessary for the diagnosis to assess whether or not the symptoms are produced intentionally, as the assessment of conscious intentionality is unreliable. The neurological examination is the fundamental tool for the diagnostic approach, being even more enlightening than the complementary tests. Hoover’s sign, Babinski’s combined leg flexion, plantar flexion of the ankle, tremor and its distraction and synchronisation manoeuvres, as well as the clinical differences between epileptic seizures and non-epileptic seizures of psychogenic origin, are some of the reliable tools for a correct diagnosis.

**Conclusions:**

The diagnosis of the disease should be one of exclusion. There must be clinical data showing clear evidence of incompatibility with a neurological disease and conversion symptoms do not correspond to known physiological mechanisms and anatomical pathways.

**Disclosure:**

No significant relationships.

